# Beneficial effects of bio-fabricated selenium nanoparticles as seed nanopriming agent on seed germination in rice (*Oryza sativa* L.)

**DOI:** 10.1038/s41598-023-49621-0

**Published:** 2023-12-15

**Authors:** Jyotsna Setty, Sanjib Bal Samant, Mayank Kumar Yadav, M. Manjubala, Vijai Pandurangam

**Affiliations:** 1 Department of Plant Physiology, Institute of Agricultural Sciences, Banaras Hindu University, Varanasi, 221005 Uttar Pradesh India; 2 Department of Mechanical and Industrial Engineering, Tallinn University of Technology, Tallinn, Estonia; 3 Department of Farm Engineering and Agricultural Statistics, Institute of Agricultural Sciences, Banaras Hindu University, Varanasi, 221005 Uttar Pradesh India

**Keywords:** Biochemistry, Chemistry, Nanoscience and technology

## Abstract

Climate change and increasing population pressure have put the agriculture sector in an arduous situation. With increasing demand for agricultural production overuse of inputs have accentuated the negative impact on environment. Hence, sustainable agriculture is gaining prominence in recent times with an emphasis on judicious and optimum use of resources. The field of nanotechnology can immensely help in achieving sustainability in agriculture at various levels. Use of nutrients and plant protection chemicals in nano-form can increase their efficacy even at reduced doses thus decreasing their pernicious impact. Seed priming is one of the important agronomic practices with widely reported positive impacts on germination, seedling growth and pathogen resistance. In the current study, the effect and efficacy of selenium nanoparticles synthesized using phyto-extracts as a seed priming agent is studied. This nanopriming enhanced the germination, hastened the seedling emergence and growth with an increase in seedling vigour and nutrient status. This eco-friendly and economical method of synthesizing nanoparticles of various nutrient minerals can optimize the resource use thus helping in sustainable agriculture by reducing environment damage without compromising on efficacy.

## Introduction

Rice is an important and staple food crop for approximately half of the world’s population^1^. It provides between 52 and 76% of the total calories that people in Bangladesh, Cambodia, Indonesia, Myanmar, and Vietnam consume in their diets, and it provides 30% of the calories that people in India and China consume^2,3^. The demand for rice will expand dramatically in the upcoming decades as the world's population is predicted to reach 10 billion by the end of 2050^4^. Cereal output will need to significantly increase in order to feed the world's expanding population and preserve food security^5^.

In the current scenario of global climate change coupled with increasing population require enhancement of crop production and productivity. Rapid seed germination, seedling establishment and growth, thus is of great contemporary importance for achieving such objectives. The time-consuming conventional breeding approaches and the still contentious genetic manipulation may not be adequate to develop varieties with enhanced germination and plant establishment^6^. Hence, physiological practices aiming to aid in plant establishment such as seed priming with various priming agents has been a proven utility in improving seed germination and seedling vigour even in the existing high yielding varieties thus, preparing the plants to tackle multiple stresses in the field more efficiently^7^. Moreover, it improves the plant biochemical status by induction and de novo synthesis of hydrolytic enzymes and improving soluble sugar levels during germination process. Nonetheless, the efficacy of each priming agent varies widely and is dependent on its intrinsic property and target crop species thus, requiring optimization specific to the need^8^. Although, the use of metal-based NPs as a priming agent is not new in agriculture, but it is becoming popular in recent times due to the tremendous benefits associated with using inorganic sources as nutri-primming agents. Nanotechnology is one of the emerging and ever-growing fields with extensive utility in various fields. Nanoparticles (NPs) are the ultrafine particles having a size range of 1–100 nm and have high surface to volume ratio which empowers them with unique physical and chemical properties. Use of such materials for modulating plant growth and physiological response is of great interest in the field of nanotechnology. The smaller size of NPs aids in rapid internalization thus helping them to affect the internal physiology and metabolism. NPs can even modulate the gene expression changing the physiology of crop plants thus affecting productivity^7^. For sustainability, applications of NPs in agriculture needs to be economical, ecofriendly, biocompatible and non-toxic thus, synthesis of bioengineered NPs for agricultural use satisfies these requisites. Plant based materials provides a sustainable alternative for the chemical synthesis methods with numerous added advantages such as non-toxic phytochemical constitutes, biochemical diversity of plant extracts, non-pathogenicity, low cost and flexibility in reaction parameters.

Various metal-NPs such as Fe, Ag, Zn, Mn, and Cu are reported to have positive effect on crop growth when used as pre-sowing agents^9^. Till date although the proof of essentiality for selenium (Se) in plants is not established, there exists numerous reports depicting its beneficial effects on plant growth. In plants, Se as a constituent of various proteins, has been reported to enhance starch and ATP synthesis^10,11^, regulate water status, delay senescence^12^ and prevent chlorophyll loss during drought. In addition, it is also reported to regulates redox reactions^13^ and cellular redox homeostasis^14^. Se is essential for human nutrition and have important physiological functions. Thus, application of Se to crops will not only benefit the plants but also eventually help in human nutrition. Due to the extremely narrow range of physiological relevance, application of Se needs to be optimum and application via SeNP provides an excellent alternative. Biocompatible SeNPs have been synthesized utilizing a green method employing rain extract from *Vitis vinifera* in an effort to support sustainable nano-agriculture.

The beneficial effect of nutripriming by nano-materials on seed germination and seedling establishment is not widely studied. Hence in this work, an alternative to traditional hydropriming will be explored: using these phytosynthesized NPs as a nanopriming agent to enhance germination and metabolic activity of germinating rice seeds of genotype HUR-105 (Malviya Sugandhit Dhan 105).In addition, rice seed can benefit from improved Se acquisition by nanopriming, increasing its nutritional value and promoting quick germination and seedling establishment. The main objectives of the current study is (1) To achieve enhanced germination and better quality seedlings for transplanted as well as direct seeded rice by using green SeNP as priming agents synthesized sustainably by using raisin extract. (2) To measure the effect of SeNP priming on germination indices including germination percentage, imbibition, seed viability and early seedling growth parameters. (3) To measure the changes inbiochemical composition like sugars, starch and α-amylase enzymatic activity via quantitative and spectroscopic methods and antioxidant enzyme activities at 24 h, 48 h and 72 h, to underline the “ROS signalling” ensuring speedy germination.

To our knowledge, a number of research have been carried out to examine the impact of Se on plant growth whether it is in bulk or in salt form; however, this is the first study of its kind examining the impacts of green-synthesized SeNPs on the morpho-physiological and biochemical changes taking place during rice seed germination. This can help farmers to prepare nano-priming materials sustainably and establish rice seedlings with enhanced vigour.

## Materials and methods

### Synthesis of SeNPs by using Raisin extract

The experiment was conducted at the Department of Plant Physiology, Institute of Agricultural Sciences, Banaras Hindu University, Varanasi, UP, India. The raisins of *Vitis vinifera* L. were procured from local market (Sahyadri Farmers Producer Company Ltd., Nashik, Maharashtra, India). Various concentrations of sodium selenite starting from 10 to 30 mM were mixed with raisin extract (Supplementary material section M1) for synthesis of SeNPs. The method described by Sharma et al.^15^ is followed with some modifications.

### Phytochemical composition analysis of Raisin extract

The presence of key phytochemicals in *Vitis vinifera* L. raisin extract was examined using the prescribed testing procedures. Preliminary analysis for alkaloids^16^, terpenoids^17^, steroids^18^, phenols^19^, saponins^20,21^, flavonoids, tannins^21^, reducing sugars, soluble carbohydrates, proteins and antioxidants^22^ were performed.

### Characterization of green SeNPs

UV–Visible absorption spectra of extract, Na_2_SeO_3_ and SeNPs was recorded in wavelength range between 300 and 600 nm using the wavelength scan function with the help of UV–Vis spectrophotometer(Labtronics LT-2201, India).The particle size and shape of SeNPs was revealed by Field Emission Scanning Electron Microscopy (FE-SEM) examination (EVO SEM MA 15/18, Carl-Zeiss, Germany).The energy dispersive X-ray spectroscopy (EDX) was performed using a Bruker EDX spectrometer to determine the elemental composition of the SeNPs.The Raman spectroscopy was conducted at room temperature at 633 nm line of laser in the spectral range of 100–500 cm^−1^ with the acquisition interval of 5 s via LabRAM HR Evol (HORIBA Scientific, USA). The FT-IR spectra of the SeNPs was determined using the Spectrum3 FT-IR spectrophotometer (Perkin-Elmer, USA) in transmission mode. X-ray diffraction (XRD) analysis was performed on high Resolution X-ray diffractometer. The pattern for XRD spectra was recorded at 2θ = 10–80^◦^ by using Cu Kα radiation (λ = 1.540593 Å) at 20 kV.

### Preparation of priming solution and seed priming method

The green synthesized SeNPs were used for seed priming and germination test. Two concentrations of SeNPs (20 µM and 25 µM) were prepared by dispersing NPs in ultra pure water via sonication for 40 min, designated as SeNP 20 and SeNP 25, respectively. Two concentrations Na_2_SeO_3_ (10 µM and 20 µM) were prepared by dissolving the salt in distilled water, designated as Se 10 and Se 20, respectively. These optimum concentrations were chosen based on the result of previous screening studies (data not shown).Healthy and uniform size rice (*Oryza sativa* L*.*) seeds were selected and were first washed thoroughly with running tap water. Subsequently seeds were surface sterilized with 2% of sodium hypochlorite for 10 min then washed with autoclaved distilled water 4–5 times to remove all the traces of the surface sterilant that may interfere with germination. Sterilized seeds were then soaked in distilled water T1-hydroprimed along with different treatment concentrations of T2—Se 10, T3—Se 20, T5—SeNP 20 and T5—SeNP 25 solutions, in the ratio of 1:5 w/v respectively for 24 h and later surface-dried on paper towel under shade. Seeds were dried back to their original moisture content at room temperature (25 ± 2 °C), sealed in cotton bags and stored at room temperature until further use. Seeds soaked in distilled water, defined as hydropriming was designated as control.

### Seed imbibition, germination test, seedling vigour and biochemical assay

The seeds of rice genotype HUR-105 (Malviya Sugandhit Dhan 105)were procured from the Department of Genetics and Plant Breeding, Institute of Agricultural Sciences, Banaras Hindu University, Varanasi. The experiments were conducted at the Department of Plant Physiology, Institute of Agricultural Sciences, Banaras Hindu University, Varanasi, UP, India. All the chemicals and solvents used in the present study were of AR grade and obtained from E. Merck (India) Ltd. To determine seed imbibitions rate, rice seeds (500 mg) were sampled and placed in distilled water for 24 h for imbibitions. Weight after imbibition was recorded after careful blot drying of water molecules adhered to the surface of seeds. Percentage imbibition was calculated as {(W2 − W1) /W1} × 100), where W1 is the initial weight of seeds and W2 is the weight of seeds after 24 h of imbibition. All the tests on germination were performed in four replications.

Twenty five seeds of each treatment were dark incubated in seed germinator at 30 °C in petri-dishes, moistened with 5 mL of distilled water. After 24 h of incubation, germination tests, α-amylase activity, total soluble sugar and starch contents were determined until 96 h at the interval of 24 h. Simultaneously, separate batches of seeds were allowed to germinate and the germination was monitored for next 7 days till 100% germination achieved in control. When the radicle length of germinating seeds extended up to 2 mm in length, the seed was considered as germinated and data on germination percentage (G%) was calculated according to previous literatures^23,24^. Further germinated seedlings were grown in modified Hoagland solution in a growth chamber for 10 days to observe the growth and vigor of seedlings under various treatments used in present study.

Viability test of seeds was performed with 2,3,5-Triphenyltetrazolium Chloride (TTC) staining, which is a frequently used method to determine the viability of cells or tissues. Red colour develops in viable tissues as a result of reaction between TTC substrate and dehydrogenases while dead tissues remain unstained.

After incubation in water, SeNPs and Se salts for 24, 48, 72 and 96 h, twenty five whole seeds with embryo intact were selected from each treatment and stained with 0.5% TTC at 35 °C for 3 h and then washed five times with distilled water and photographed under a SteREOLumarV12 stereomicroscope^25,26^.

Total soluble sugars (TSS) were estimated using anthrone reagent as per the procedure outlined by Dubois et al.^27^. Starch content was estimated using the method described by Hodge and Hofreiter^28^. The activity of α-Amylase enzyme was assayed from the saccharifying activity as per the protocol described by Bernfeld^29^. The enzyme activity calculated by the formula given by Yaldagard et al.^30^. Qualitative assessment of the effect of Se priming (Na_2_SeO_3_ and SeNP) on α-amylase activity, with starch plate test using embryoless half-seeds according to the method of Chen et al.^31^.

### Superoxide dismutase, catalase, and ascorbate peroxidase activity measurements

A crude extract of the enzymes from rice seed embryos was prepared and the activities of SOD, CAT and APX were determined spectrophotometrically^32,33^. The mean value ± SE for each experiment of four biological replicates were calculated.

### Histochemical localizationin seeds

The accumulation of free radicals H_2_O_2_andO_2_^−^ and were assessed using 3,3-diaminobenzidine (DAB), nitro-blue tetrazolium (NBT) respectively^34^ and peroxidase activity was analyzed by 3,3′,5,5′-Tetramethylbenzidine (TMB) staining in seedsand all samples were photographed with the SteREOLumarV12 stereomicroscope.

### FTIR functional characterization and EDX elemental composition analysis of primed seeds

To analyse the effect of priming on various functional groups (biomolecules) present in seeds, FTIR was performed. Small beads were prepared by mixing dehulled rice grain powder (2 mg) with KBr (1:100 p/p) for FTIR spectroscopy (Perkin-Elmer model) and spectra were recorded between wavenumbers400 and 4000 cm^−1^.

Elemental composition analysis was done to investigate mobilization and accumulation of SeNPs in the rice seeds. The concentrations of macronutrients such as, C, O, N, P, K, Ca, Mg, S and micronutrients such as Fe, Zn Cu, Mn and beneficial elements such as, Se, Na and Si in the seeds were detected by an energy dispersive X-ray analysis (EDX-Microanalysis, INCA). The content of elements was expressed in % weight basis.

### Statistical analysis

The data obtained from four replicates were analysed for Analysis of variance (ANOVA) and Duncan's multiple range test (DMRT) post-hoc analysis by using R (v 4.2.1).

### Ethical statement

We have, to our best knowledge, complied with the existing institutional and national guidelines and with the Convention on Biological Diversity and the Convention on the Trade in Endangered Species of Wild Fauna and Flora. The plant materials used in the experiment are Rice genotype HUR-105 (Malviya Sugandhit Dhan 105) procured from the Department of Genetics and Plant Breeding, Institute of Agricultural Sciences, Banaras Hindu University, Varanasi, and the raisins of *Vitis vinifera* L. used were procured from the local market kindly provided by Sahyadri Farmers Producer Company Ltd., Nashik, Maharashtra, India.

## Results

### Phytochemical screening analysis

The raisin extract of *Vitis vinifera* L. was analysed for its phytochemical constituents. Phytoextracts often contain several biochemical compounds such carbohydrates, proteins, terpenoids, tannins etc. Alkaloids, flavonoids, saponins, phenols, terpenoids, and antioxidants are the main secondary metabolites found to be present in the raisin extract, as shown in Table 1 (Supplementary). It has been revealed that –OH groups present in phenols and flavonoids play important role in providing stability to SeNPs synthesized from selenite via bio-reduction. Furthermore, groups of heterocyclic compounds such as C=O, C=C and C=O–C also help in stabilizing the phytochemically synthesized SeNPs^35^.

### Characterization of green synthesized SeNPs

**Figure 1:**
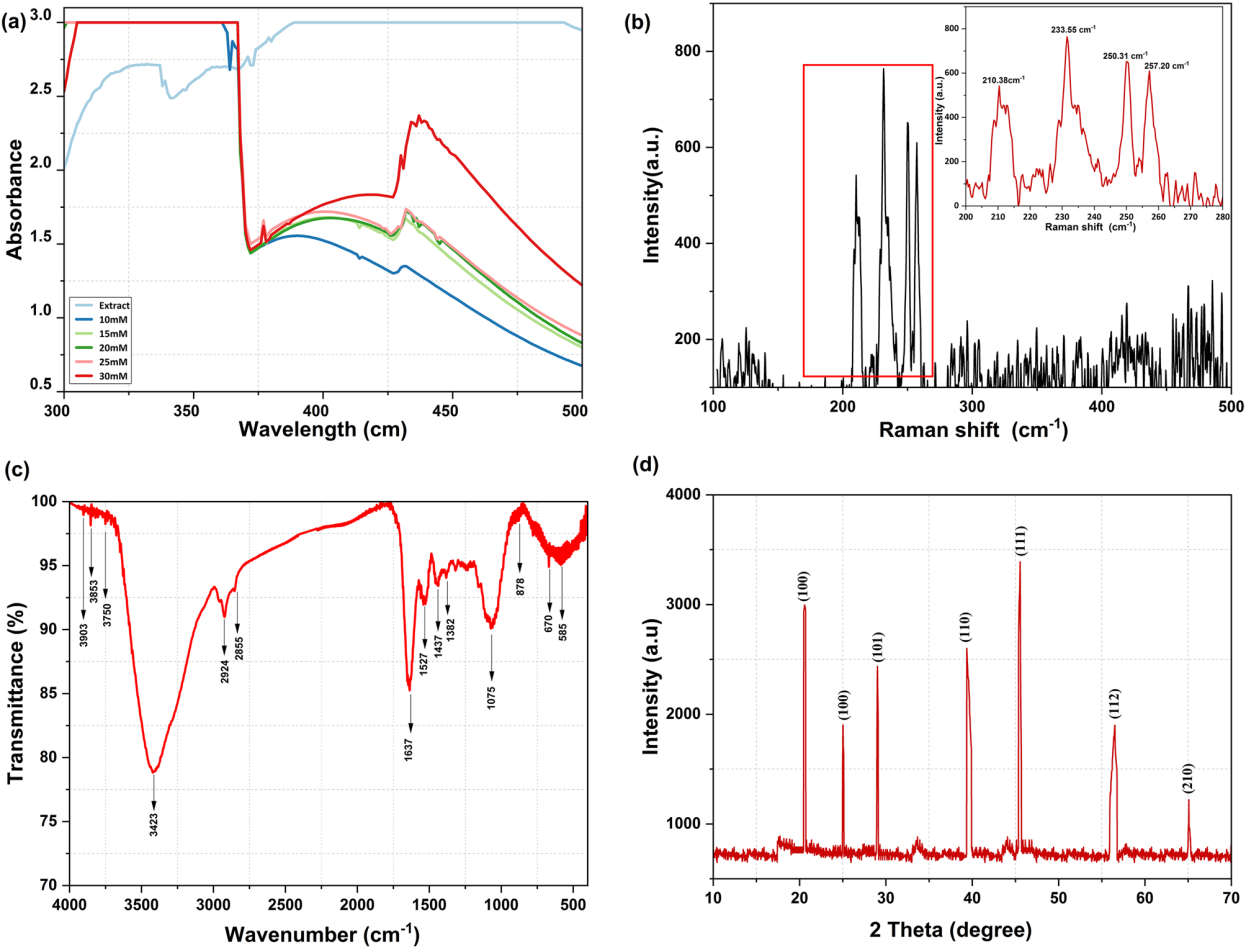
Characterization of green synthesized SeNPs (**a**) UV–visible spectrum (**b**) Raman spectrum (**c**) FTIR spectrum (**d**) XRD spectrum.

During the green synthesis process, reaction between Sodium selenite (Na_2_SeO_3_) and reducing agents present in *Vitis vinifera* raisin extract releases Se ions which converts to the SeNPs. The validation of the same can be assessed from the visual colour change in which the pale yellow colour of the solution changes to dark red ensuing the synthesis process. UV–Vis spectroscopy is another preliminary test performed to confirm the synthesis of SeNPs. Figure 1a depicts the absorbance in the range of 300–500 nm wavelength with maximum absorption in the range of 400–450 nm and a strong peak at 430 nm, illustrating the formation of SeNPs in the reaction mixture. The peak shifted towards the blue region in lower concentration (10 mM) depicting nanoparticles of lower diameter but the yield remained comparatively low as observed from lower absorbance. At high concentration of 30 mM the secondary peak became prominent owing to the aggregation of the particles which is also clear from the higher absorbance in the red region of spectrum (500 nm), hence 25 mM was found to be the optimum concentration for the present protocol and the particles obtained from the concentration were used for further characterization and study.

Raman spectroscopy provides insights into various allotropic modifications and crystallinity and obtained spectrum support the crystalline nature of synthesized NPs as it confirms formation of trigonal selenium (t-Se) by the appearance of resonance peak at 233.55 cm^−1^ which shows that crystallization has occurred (Fig. 1b) and justifying the previous reports^36–42^. A sharp peak at 250.31 and 257.20 cm^−1^ is a characteristic absorption band represents monoclinic Se (m-Se) and A1 mode amorphous Se (A1 stretching Se–Se mode)^43^.

FTIR spectroscopy was performed for determination of the surface structure of the SeNPs and the phytochemical functional groups attached to it. Figure 1c shows a broad peak obtained at 3423 cm^−1^ corresponding to the hydroxyl group vibration (–OH) of alcohols and phenols. Other weak peaks obtained at 1527, 1437 and 1382 cm^−1^ represent aromatic nitro compound, N–O stretching and alkyl ethers respectively. Stretching vibration corresponding to peak 1637 cm^−1^ represents typical protein bands of amide I. Peak at 1074 indicate vibration between N–H, whereas 878 and 670 signifies C–X stretching in alkyl halides, and carboxyl group respectively^44–51^. These groups not only help in the reduction process during the synthesis of SeNP but also help in stabilization of the particles.

Figure 1d shows the XRD spectra of the synthesised SeNPs. The distinctive peaks were detected corresponding to 2θ values = 20.58° (100), 25.02° (100), 29.05° (101), 39.36° (110), 45.54 (111), 56.5 (112) and 65.08 (210) of the SeNP that support the crystalline nature. The planes at (101) and (110) indicate the spherical and oval nature of NPs formed. Size of NPswas found to be averaging around 40 nm as determined by Debye–Scherrer equation. Crystallinity of the synthesized SeNPs can be inferred from the sharp and narrow peak^44^.

**Figure 2:**
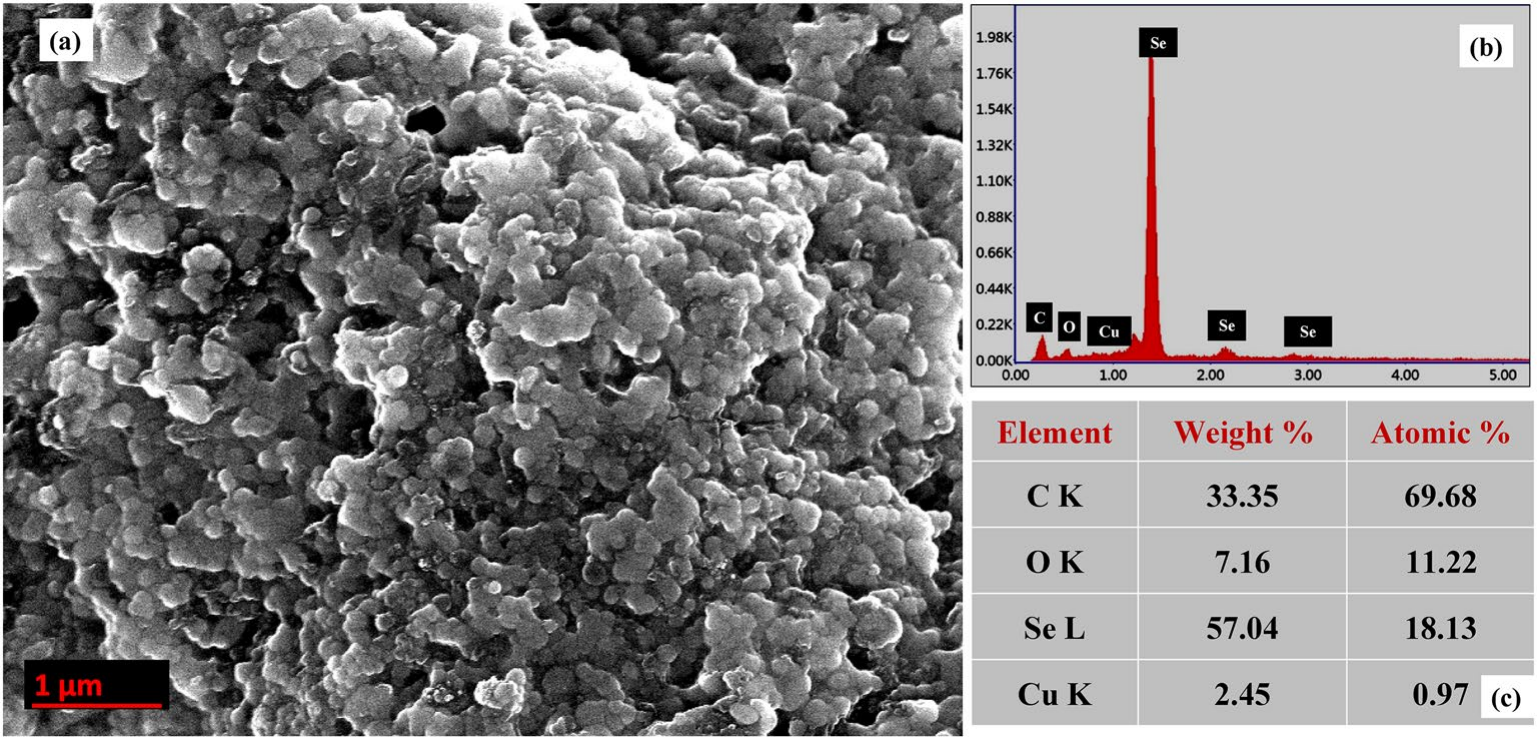
Morphological characterization of SeNPs (**a**) SEM image of SeNPs at 1 µm (**b**) EDX spectrum and elemental composition of SeNPs.

The morphological characters of synthesized NPs were determined by SEM analysis. Images of SEM obtained at resolution of 1000 nm (Fig. 2a) demonstrated the presence of irregular to oval and near-spheroid shaped SeNPs with smooth surfaces. EDX analysis conducted to determine the elemental composition of the NPs (Fig. 2b)showed a strong signal in the Se regions representing the formation of SeNPs and weak signal in C, O and Cu region due to the biomolecules present in NPs coating, with a composition of 57.04% Se, 33.35% C, 7.16% and 2.45% O.

### Effect of priming on germination rate

**Figure 3:**
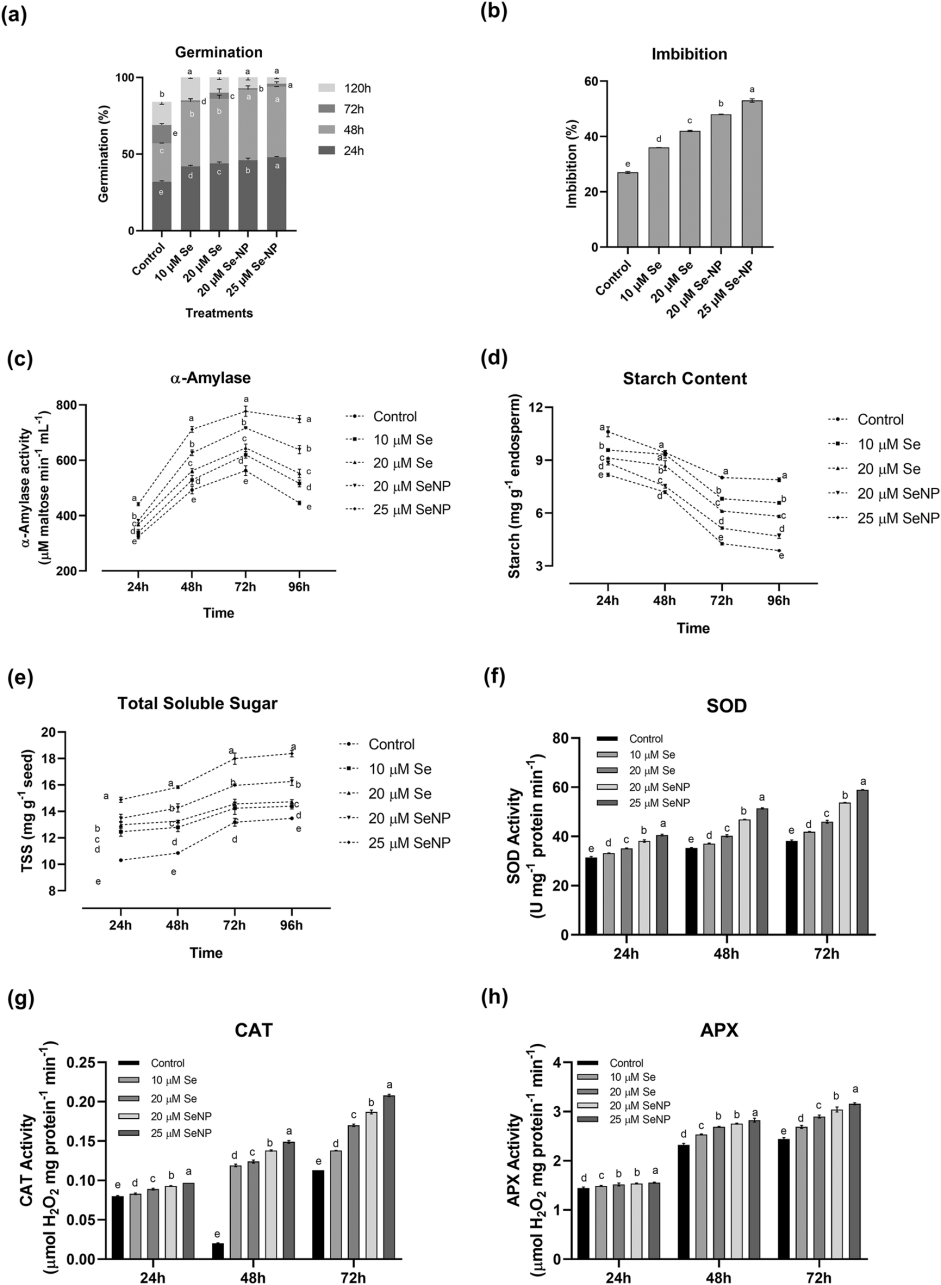
Physiological parameters of germinating rice seeds. (**a**) Germination% (**b**) Seed imbibition% (**c**) α-amylase activity (**d**) Starch content (**e**) Total soluble sugar content (**f**), SOD activity (**g**) CAT activity and (**h**) APX activity. All values are the mean of four replicates (± SD). ANOVA significant at (p < 0.01). Different letters indicate significantly different values in a particular tissue (DMRT, p < 0.01).

Rice seeds primed with SeNP 20 µM and SeNP 25 µM (shown in Fig. 3a) showed early protrusion of radicles thus enhanced germination compared to hydroprimed control. Nanoprimed seeds attained germination up to ~ 95% after 48 h of incubation and also showed significantly higher rate of germination than the selenite primed seeds (Se 10 µM, Se 20 µM). Even though selenium treatments as selenite and nanoparticle form were not significantly different in improving the rate of germination, they were significantly higher from hydroprimed control treatment. In the present study both the nanoprimed and Se-salt primed seeds achieved 100% germination faster (5 days) compared to control (7 days) which indicates that nanopriming play important role in early emergence of radicle leading to early seedling establishment.

### Effect of priming on starch mobilization

The activity of the α-amylase, as well as the total amount of soluble sugars and starch content, were tested to ascertain the biochemical alterations in starch metabolism brought on by nanopriming. α-amylase is essential for the metabolism of carbohydrates; it hydrolyzes stored carbohydrates into soluble sugars to enable resumption of metabolism and subsequent growth until the development of photosynthesizing green tissue^52^. Nanopriming with SeNPs significantly enhanced α-amylase activity in germinating rice seeds compared with other priming treatments (Fig. 3c). The rate of change in α-amylase activity with respect to time, showed significant escalation in the tune of ~ 65% in seeds primed with 20 µM SeNP followed by 60% in seeds primed with 25 µM SeNP concentration after 48 h of imbibition compared to selenite treatments and hydroprimed control (Fig. 3c). Even though, higher enzyme activity was observed in seeds primed with 25 µM SeNP after 72 h of imbibition which was measured in terms maltose produced by the α-amylase (777.3 μM maltose min^−1^ mL^−1^), the significant augmentation was noticed after 48 h itself as result of increased rate of starch degradation leading to high sugar content (discussed below). The current findings are consistent with earlier research that suggested improved starch mobilization in response to priming^53,54^.

**Figure 4:**
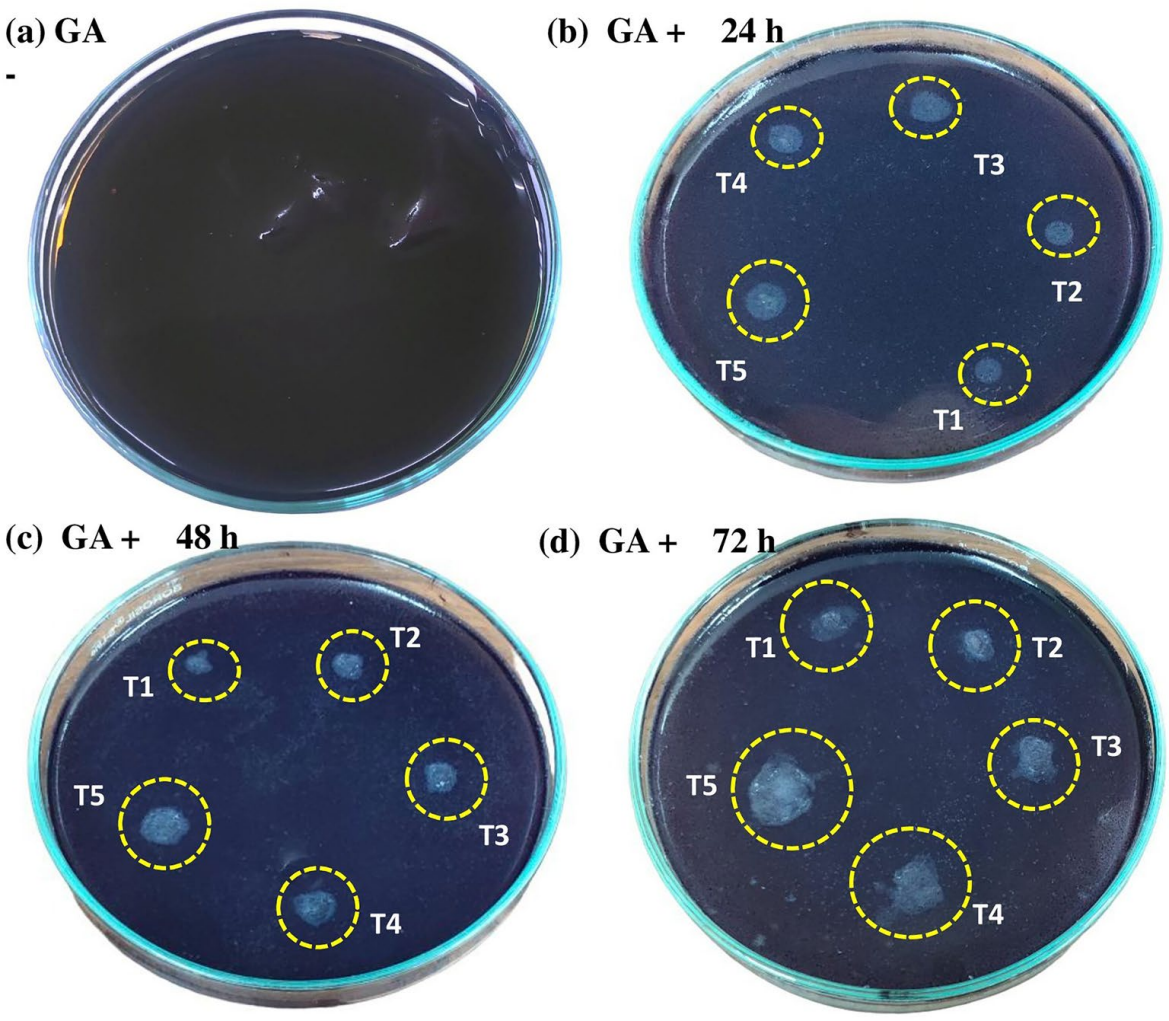
Starch agar plate assay for detecting α-amylase production of different primed seeds compared with control. Clear zone indicates amylase production.

According to Man et al.^55^ enhanced α-amylase activity in nanoprimed seeds after 24 h of imbibition can be attributed to the entry of NPs to the seeds thus eroding starch surfaces and providing avenues for the hydrolyzing enzymes to act upon. This coupled with increased amylase activity has the potential to cause rapid starch breakdown, which would result in higher soluble sugar content thus, enhancing seed germination and seedling growth in nanoprimed seeds. This result was further confirmed by analyzing α-amylase production and activity from embryoless half-seeds using a starch agar plate α-amylase assay method in different primed seeds. Clear zones appeared in starch agar plates around the incubated half-seeds after IKI staining (Fig. 4b–d), indicating secretion of α-amylases.After an incubation of 3 days, larger and prominent clear zones around nanoprimed seeds are observed indicating an increased production of α-amylase from nanoprimed seeds than other priming treatments.

Starch is the major source of energy for rice embryos, and amylase activity is responsible for mobilization of starch reserves i.e. breakdown of starch into simple sugars^56^. The reserve starch in seeds decreased with time (Fig. 3d) and lower starch content was observed in seeds primed with 25 μMSeNP which was 3.87 mg of starch g^−1^ endosperm^−1^. Similarly, total soluble sugar content was estimated to corroborate the effect of starch hydrolysis. Interestingly, the rate of reduction instarch content was noticeably higher in nanoprimed seeds but without corresponding increase in total soluble sugars (Fig. 3e). The results indicate significantly higher sugar content in nanoprimed seeds compared to other treatments (Fig. 3e) during all imbibition periods used in experiment where the maximum value observed was 18.37 mg g^−1^ in 25 µM nanoprimed seed after 96 h of imbibition. Interestingly, when observed with respect to growth duration (Fig. 3e), hydroprimed (control) seeds showed 21% increase in the TSS content after 48 h of imbibition which is comparatively higher compared to nanoprimed seeds and selenite primed seeds which showed only 14% and 11% increment respectively in same duration. This could be a consequence of increased utilization of produced sugars by the germinating nanoprimed seeds as this showed higher vigour.

### Effect of priming on embryo viability and seedling vigour

**Figure 5:**
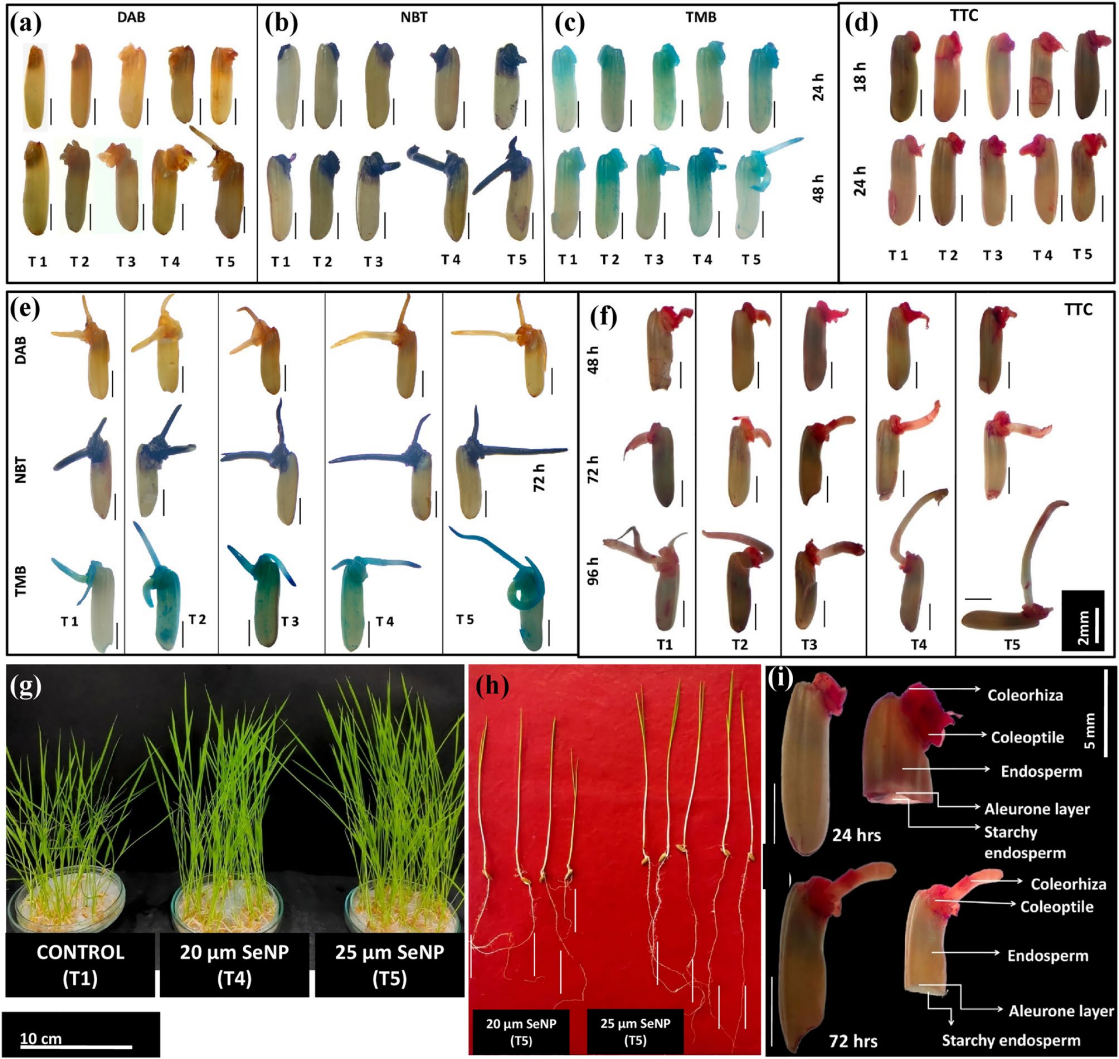
Effect of different concentrations of SeNPsnanopriming at different intervals on (**a**) superoxide generation using NBT staining (**b**) hydrogen peroxide generation using DAB staining (**c**) Peroxidase activity was assessed by staining with TMB in rice seeds imbibed for 24 and 48 h and (**e**) for 72 h respectively (**d**) seedling vigor by TTC staining in rice seeds imbibed for 18 and 24 h and (**f**) for 72 h respectively. (Scale bar = 2 mm). (**g**) Seedlings after 10 days of germination (Scale bar = 10 cm). (**h**) Seedlings after 3 days of germination (Scale bar = 2 cm). (**i**) TTC staining of horizontal cut section of rice seed (Scale bar = 5 mm). (T_1_ (Control), T_2_ (Se 10), T_3_ (Se 20), T_4_ (SeNP 20 and T_5_ (SeNP 25).

TTC staining is an effective and widely used method to assess seed viability. Seed imbibition leads to the activation of dehydrogenase enzymes which results in release of hydrogen ions. This mediates the reduction of colorless TTC into formazan staining living cells with a red color. To examine the effect of SeNP and Selenite on seed viability, germinating hydroprimed, selenite primed and nanoprimed seeds were stained with a TTC solution (Fig. 5d,f,i). The embryo at 18 h and 24 h (Fig. 5d), coleoptile and coleorhiza at 48 h (Fig. 5f,i) and radicle at 72 h (Fig. 5f,i) stained red, whereas the endosperm and aleurone layer of the whole seed stained minimally. The staining results showed comparable staining of embryos in all the treatments implying more or less uniform embryo viability. When the nanoprimed seeds after 24 h of incubation were horizontally cut in half, the aleurone layer also showed staining (Fig. 5i). Remarkably, after 74 h of incubation, staining of the aleurone layer of half seeds was completely negligible (Fig. 5i), but the protruding radicle showed pink formazan staining indicating stimulatory effect of dehydrogenase activity in radicles. Figure 5f shows the faint formazan stain in radicles of selenite primed and hydroprimed seedlings compared to nanoprimed seedlings. Also, the length of radicle was significantly higher in nanoprimed seedlings compared to other primed seedlings after 96 h of incubation showing early emergence of radicle. This data was in accordance with the faster rate of germination in nanoprimed rice seeds in comparison to selenite and hydroprimed control. It has been earlier reported that rice seedlings primed with zinc NP exhibit robust plumule and radicle growth along with significantly higher biomass as compared to hydroprimed seedlings^9^ further supporting the current findings.

### Effect of nanopriming on ROS status in rice seeds

ROS are critical for seed germination. In this study staining dyes i.e., DAB and NBT were used for histochemical localization of ROS species viz. H_2_O_2_and O2^·–^ accumulated during rice seed germination. After staining with DAB, the nanoprimed seeds showed higher accumulation of dark brown spots in the coleorhiza and radicle whereas aleurone layer was stained with pale brown colour compared to other treated seeds especially by 48 h (Fig. 5a,e) showing an increased level of H_2_O_2_ accumulation. However, the staining intensity in the coleorhiza and radicle was reduced after 48 h and especially after 72 h of germination, in all treated seeds. The staining of coleoptiles of nanoprimed seedlings after 72 h of germination with pale brown colouration (Fig. 5e), depicts the involvement of H_2_O_2_ in the processes of cell elongation. By comparison, the coleoptiles from selenite and hydroprimed seedlings were stained with similar intensity.

When seeds stained with NBT, a dark purple-blue colour stain observed after 24 h of germination, at the time of coleorhiza emergence. The coleorhiza of nanoprimed seeds showed increased accumulation of O2^·–^compared to other treated seeds (Fig. 5b). The stain became darker in the embryo and radicle after 48 h (Fig. 5b), showing critical role of O2^·–^ in protrusion of radicle in all primed seeds. In addition, the aleurone layer of the whole seed was not stained this might be due to either O2^·–^ not being produced in outer cells of the aleurone layer. The coleoptile of the seeds showed very strong staining showing higher production O2^·–^, suggesting its important role in the emergence of coleoptile.

Due to the shorter half-life of^•^OH, it is hard to directly quantify^57^. In order to evaluate the ^·^OH generation, indirect measurement in terms of peroxidase activity can be utilized^58^. When germinating primed seeds were treated with TMB, the staining intensity of embryo and endosperm were stronger compared to the staining intensity of NBT and DAB. As shown in (Fig. 5c), unlike the O2^·–^ and H_2_O_2_ staining, the endosperm showed more visible staining. Meanwhile, the intensity of TMB staining in the embryo was proportional to the imbibition time, especially in the coleorhiza, radicle and coleoptile. Notably, the coleoptile also showed strong staining, similar to what was observed for O2^·–^, suggesting the converging role of O2^·–^, ^·^OH in coleoptile emergence. The result shows that in nanoprimed seeds, the intensity of TMB staining in the endosperm, embryo, coleoptile and especially in the coleorhiza was higher after 72 h (Fig. 5e) relative to selenite and hydroprimed seeds, indicating the SeNP mediated increased the peroxidase activity of germinating seeds.

### Effect of nanopriming on antioxidant enzyme activity in germinating seeds

In order to combat oxidative stress, plants detoxify ROS by up-regulating antioxidant enzymes such as SOD, CAT, peroxidase (POX), APX, etc. alongside non-enzymatic antioxidant compounds (ascorbate, glutathione, etc.)^32,58^. These antioxidant enzymes are essential for scavenging ROS in plants, but intriguingly, they also play a substantial role in preventing ROS generation in the first place by serving as a positive regulator in plant seed physiology^59^. The results shown in (Fig. 5a–e) indicated that SeNPs enhanced the accumulation of ROS in embryo of seeds, but to understand its effect(s) on ROS-degrading enzymes, the activities of CAT, SOD, and APX, were examined in primed seeds. The activities of SOD, CAT and APX showed gradual increase and peak at 72 h (Fig. 3f–h), during germination in all primed seeds. For seeds primed with SeNP, the SOD and CAT activities increased, especially between 24 and 48 h of germination, whereas, 25% stimulation in SOD activity (Fig. 3f) and more than 50% and 80% enhancement in CAT (Fig. 3g) and APX activity (Fig. 3h) respectively were observed in nanoprimed seeds between 24 and 48 h. Further this increment was 15%, 40% and 75% in selenite primed seeds and 12%, 25% and 60% in hydroprimed seeds between 24 and 48 h. Above discussed results shows the altering activities of enzymes in seeds with respect to time but when their performance was analyzed among different treatments, enhanced activities of SOD and CAT in nanoprimed seeds is observed compared to hydroprimed and selenite primed seeds while APX activity essentially remains unchanged in all treatments. SOD, CAT, and APX are ROS-degrading enzymes and are involved in the redox homeostasis in plants^32^. SOD and catalase acting in tandem scavenges O2^·–^ toH_2_O_2_ and finally to H_2_O^60^. APX via glutathione-ascorbate cycles helps in scavenging of H_2_O_2_ into water utilizing ascorbate as the electron donor^61^. SeNPs increased the SOD and CAT activities, especially after 48 h of germination, but had no substantial effect on APX activity, indicating the specific role of SeNPs in enhancing the activity of SOD and catalase thus maintaining the redox balance in the embryo. These results are in consonance with earlier studies where differential activity of SOD and CAT antioxidant enzyme is observed in unprimed and primed seeds in response to different seed priming treatments using green synthesized silver NPs^62^. Sharma et al.^63^ demonstrated altered activity of antioxidant enzymes (SOD, CAT, and APX) in rice seedling treated with different concentrations of molybdenum NPs (α-MoO3 or MoS2 NPs). Similarly, Sharma et al.^9^ used green synthesized zinc NPs by *Senna occidentalis* leaf extract as priming agent in aged rice seeds of BM6 of Pusa basmati (early flowering homozygous mutant) and observed substantial increase in antioxidant enzyme activity.

### Functional characterization of seeds by FTIR spectroscopy

**Figure 6:**
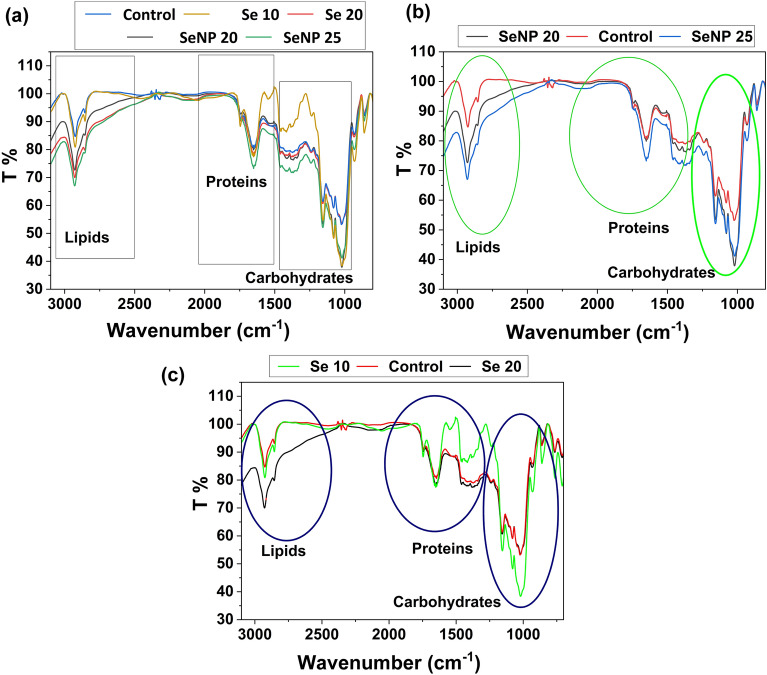
FTIR of rice seeds with different priming treatments.

To get better understanding of biochemical changes on primed seeds, estimation of differential alteration in metabolites in all primed seeds was performed using FTIR (Fig. 6). The IR graph shows sharp absorption peaks in the mid-IR region (2000–1000 cm^−1^) and end region (3000–2800 cm^−1^) indicating accumulation of carbohydrates, proteins and lipids in all primed seeds (Fig. 6). FTIR spectra showed minor increase in nanopriming induced carbohydrate accumulation in both the concentrations of NPs used compared to other selenite primed and hydroprimed seeds (Fig. 6a). Similarly in regions corresponding to proteins, an increase in band intensity was observed, depicting a considerable accumulation of proteins in nanoprimed seeds when compared with hydroprimed seeds (Fig. 6b), while this increase was more or less similar in selenite primed seeds (Fig. 6c). The absorption peaks corresponding to protein is generally located between 1800 and 1500 cm^−1^ and consisted of amide-I and amide-II^64^. The IR spectrum corresponding to lipids mainly occurred between 3000 and 2800 cm^−1^ and IR peaks were observed in this region indicating a significant increase in lipids and fatty acids in seed primed with selenite and NPs, however this increase was more prominent at nanoprimed seeds (Fig. 6a). Notably the results indicate a decreased transmittance and increased absorbance ratio of IR spectra in the carbohydrate, protein and lipids regions, in nanoprimed and selenite primed seeds compared to hydroprimed seeds which suggest that both selenite priming and nanopriming had a beneficial impact on biomolecules synthesis. Moreover, priming with SeNP modulates the cellular bio-molecular constituents positively and bring further metabolic changes which may be a contributing factor to early germination.

## Discussion

**Figure 7:**
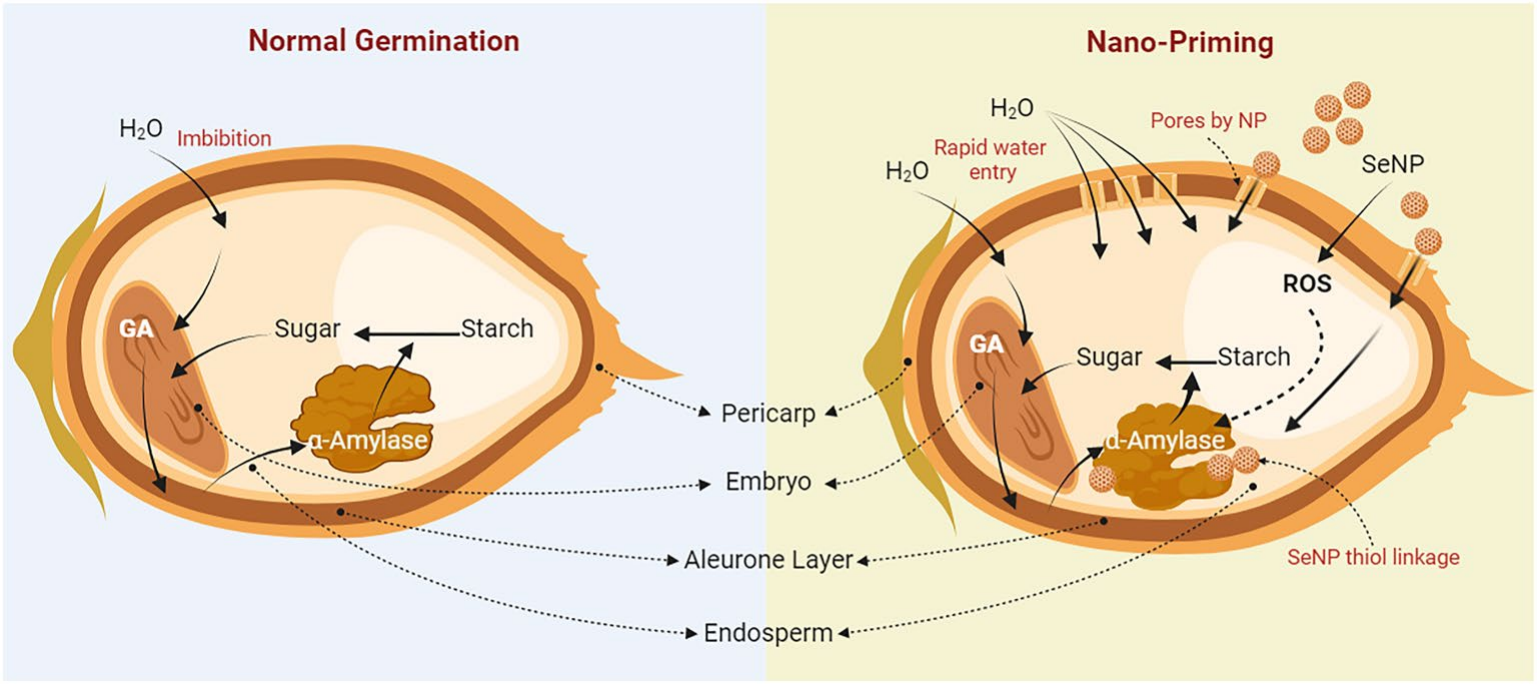
Anticipated model of SeNPs induced germination in rice by the following mechanisms. Hydroprimed seed with slow water uptake and low metabolic activity. In hydroprimedseeds the hydrolysis of starch occur gradually and thus available sugars is low in the initialphase, exhibiting slow germination. But in case of SeNPnanopriming at first,during the initial phase of imbibition, SeNPspenetrate the seed coat creating small pores and facilitating water uptake intoseeds which in turn emulsify the stored nutrients of endosperm and transport them to embryo via theepithelial cells of the scutellum after increased water uptake and up-regulation of theexpression of aquaporin genes involved water uptake. Secondly, in the second phase, the α-amylase could possibly interact with functional groups ofbiomolecular ligands on the surfaceofSeNPs through the thiol linkages as NPs-amylase complex leading to higher metabolicactivity by increasing starch hydrolysis and generating large amount soluble sugarsand hence lowering the osmotic potential and water potential. This drives more water uptakeand creates high mechanical resistance in whole tissues of cell wall causing its loosening. Thirdly, in the last stage, aquaporins facilitate H_2_O_2_ or ROS diffusion acrossbiological membranes. SeNPs mediated ROS elevation is tightly controlled by seed antioxidantsystems to maintain ROS in optimum range of oxidative window which help in its functioning as signalling molecules. The ROS participate in cell wall loosening and endosperm weakening and also trigger essentialmetabolic activity of seed for promoting seed germination.

Changing climate can subject the germinating rice seeds to a plethora of stress thus affecting its growth and developmental physiology such as germination percentage, seedling growth, vigour and early establishment. Hence, application of various seed treatment methods to enhance the germination, seedling establishment and early growth can benefit the crop immensely and translate to yield. Treatment of seeds by nanomaterials is earlier reported to enhance its resilience to biotic and abiotic stress^65^. In the current study we synthesized selenium nano-particles by using raisin extract as source of reducing agents. Characterization by various methods such as UV–Vis, XRD, FTIR, Raman, FE-SEM, and EDX confirmed synthesis of spherical SeNPs stabilized by phytochemicals (Figs. 1, 2). Synthesis of SeNPs via this method eliminates the need of any environmentally harmful chemicals as reducing agents thus making it sustainable and low cost. In order to study the efficacy of bio-synthesized SeNP as a seed nano-priming agent, rice seeds were treated with SeNP solution as a priming treatment. This priming treatment led to an enhanced germination percentage and showed earlier emergence of radicles (Fig. 3a). Wojtyla et al.^66^ reported higher efficiency of primed seeds for water uptake compared to unprimed seeds and enhanced seed imbibition. In earlier reports^9,67^, nanopriming using gold NPs and Zn NPs increased water uptake compared to conventional priming measures. In the present study after 24 h of imbibition, more than 50% stimulation of water uptake was observed in the SeNP primed seeds as evident from the imbibition % of seeds (Fig. 3b). This data further corroborates the early emergence of radicle and faster rate of germination in nanoprimed rice seeds in comparison to selenite and hydroprimed control (Fig. 3a). Thus, priming with nanoparticles causes higher water uptake in nanoprimed seeds leading to faster wetting of seed coat and its softening. Higher imbibition of water also leads to activation and release of hydrolytic enzymes involved in reserve mobilization from the aleurone layer, resulting in faster growth of embryo and its emergence out of seed coat and seedling establishment (Fig. 7). Further, an increased rate of germination can also be attributed to entry of NPs inside, or adsorption on the surface of seed coat followed by transport to inside through the spaces in the textured parenchymatous tissues. Due to their smaller size and large surface area, NPs can easily enter into the cell^68^. An increment in the germination percentage in nanoprimed seeds can also be attributed to the enhanced activity of α-amylase allowing faster hydrolysis of reserve endospermic starch. The survival and continuation of seedling growth showed positive correlation with amylase activity (Fig. 3c)^1,69^. The rapid activation of α-amylase and subsequent enhanced availability of simple sugars increased the embryo viability andseed vigour. ROS plays important role during seed germination.

ROS have far-reaching physiological consequences. ROS act as pivotal signalling molecules albeit in optimum quantity. Hence during seed germination, ROS level should be within the optimum range known as “Oxidative window” necessary for the signalling^59,70^ and germination might cease if there is any deviation from this range. H_2_O_2_ also functions as a germination-controlling signalling molecule. The antioxidant machinery regulates it within a range to strike a balance between signalling that promotes germination and detrimental effects caused by oxidative stress which hinder germination. Previous studies have illustrated the action of ROS on plant cell-wall polysaccharides. ROS aids in breaking of glycosidic bonds, which cause the cell wall to relax, thereby promoting cell division and elongation^71–73^. Dicotyledonous plants like cress and lettuce produce ROS in their micropylar endosperm and radicle during seed germination, which causes endosperm weakening, and radicle elongation thus, helping in seed germination^57^. These activities also call for certain cell-wall hydrolases as well as non-enzymatic components such as expansin^74^. ROS signalling modulates the metabolism, nutrient mobilization and other physiological processes^75^, whereas ROS can directly act on cell wall polysaccharides, further loosening the plant cell wall and eventually facilitating the germination further^76^. This study demonstrated the crucial role of SeNPs in ROS modulation and signalling which aids in enhancing the germination (Fig. 5a–c).To maintain the ROS homeostasis,activation of various anti-oxidant enzymesis important and in the present study such enzymes showed enhanced activity albeit towards the later stages of germination (Fig. 3f–h). Uptake of NPs impaired oxidative balance which in turn led to modulation of the antioxidant enzymes to maintain the redox balance. Taken together the upregulation of certain antioxidant enzymes in nanoprimed seeds leads to the fine tuning of ROS necessary for the signalling needed for metabolic reprogramming which culminates in enhanced germination and growth. In order to understand the differential modulation of cellular biochemical constituents by SeNP priming during seed germination, FTIR spectroscopy was performed. This showed enhanced accumulation of proteins, fatty acids, lipids and also corroborated the enhanced accumulation of sugars in response to nanopriming. Accumulation of these biochemical constituents can significantly enhance the malting quality of rice seeds help in early germination and seedling establishment^77^.

Taken together the current study showed the efficacy of sustainably synthesized bio-fabricated SeNPs as a seed priming agent. The priming treatment enhanced the seed germination, embryo viability and seedling vigour, Though the mechanism remains obscure, based on previous reports and current experiments a learned inference can be derived. The SeNPnanopriming leads to the development of nanopores during the entry of Se into the seed which then channels the water into the intramural parts of seed (as observed from the increased imbibition%) causing higher water uptake in such seeds compared to other. This further modulates the metabolic processes (viz. enhanced activity of amylase and antioxidant enzymes) and the differential water status becomes pivotal for early development and establishment of seedling.

## Conclusions

Nanotechnology is leading to the development of a range of inexpensive applications in the field of agriculture. To our knowledge, this is one of first of its kind studies related to the effects of green-synthesizedSeNPson morpho-physiological andbiochemical changes during rice seed germination.The economic and sustainable production technique of such nano-particles with the added advantage of enhanced nutrient status can immensely benefit the agricultural sector. This technique can be practiced to increase the seedling vigour and can also be explored by farmers and seed companies. The combination of various nano-particles for seed treatment and seedling application can also reduce the chemical needs in form of fertilizer and plant protection chemicals at all stages of crop development due to its higher efficacy, effectivity and lower dosage thus attaining the objective of sustainable agriculture.

### Supplementary Information

Supplementary Information.

## Data Availability

The datasets generated during and/or analysed during the current study are available from the corresponding author on reasonable request.
