# Supplementary material for: Beneficial effects of bio-fabricated selenium nanoparticles as seed nanopriming agent on seed germination in rice (Oryza sativa L.)
Source: Sci Rep. 2023 Dec 15;13:22349. doi: 10.1038/s41598-023-49621-0 (PMC10724239; doi:10.1038/s41598-023-49621-0)
Supplement: Supplementary file 1 — Supplementary Information. [file 41598_2023_49621_MOESM1_ESM.pdf]

Title- Beneficial effects of bio-fabricated selenium nanoparticles as seed nanopriming agent on seed germination in rice (*Oryza sativa* L.)

Authors-

Jyotsna Setty*[a], Sanjib Bal Samant[a] , Mayank Kumar Yadav[b], Manjubala M[c], Vijai Pandurangam *[a]

Affiliation

[a.] Department of Plant Physiology, Institute of Agricultural Sciences, Banaras Hindu University, Varanasi, 221005, Uttar Pradesh, India.

[b.] Department of Ceramic Engineering, Indian Institute of Technology- Banaras Hindu University (IIT-BHU), Varanasi, 221005, Uttar Pradesh, India.

[c.] Department of Farm Engineering and Agricultural Statistics, Institute of Agricultural Sciences, Banaras Hindu University, Varanasi, 221005, Uttar Pradesh, India.

Supplementary Materials

Methodology

Synthesis of SeNPs by using Raisin extract

Various concentrations of sodium selenite starting from 10 mM to 30 mM is mixed with the raisin extract (Supplementary material section M1) for synthesis of SeNPs. The method described by Sharma et al. (2014)[10] is followed with some modifications.

Phytochemical screening and spectroscopic analysis

The presence of many key phytochemicals in the *Vitis vinifera* L. raisin extract was examined using the prescribed testing procedures. Preliminary analysis for Alkaloids (Harborne, 1973; Ekwueme et al., 2015), Terpenoids (Tiwari et al., 2011; Panchal et al., 2021), Steroids (Hossain et al., 2013; Ekwueme et al., 2015), Phenols (Ali et al., 2017), Saponins (Okerulu et al., 2017; Hashmi et al., 2021), Flavonoids (Sajid et et al., 2016; Hashmi et al., 2021), Tannins (Khan et al., 2012; Hashmi et al., 2021), Reducing Sugars

(Harborne, 1973), Soluble Carbohydrates (Harborne, 1973), Proteins (Tiwari et al., 2011; Sharma et al., 2015) and Antioxidants (Sharma et al., 2015) were performed by adopting the standard procedures.

Characterization of green SeNPs

UV-Vis absorption spectra of extract, Na_2SeO_3, SeNPs were recorded with the help of UV–vis spectrophotometer by recording the wavelength range between 300–600 nm. The Raman spectroscopy was conducted at room temperature at 633 nm line of laser in the spectral range of 100 – 500 cm^{-1} with the acquisition interval of 5 seconds via LabRAM HR Evol (HORIBA Scientific, USA). The FT-IR spectra of the SeNPs, was determined using the Spectrum3 FT-IR spectrophotometer (Perkin-Elmer, USA) in transmission mode. X-ray diffraction (XRD) analysis was performed on high Resolution X-ray diffractometer. The pattern for XRD spectra was recorded at $2\theta = 10–80^\circ$ by using Cu Kα radiation (λ= 1.540593 Å) at 20 kV. The particle size and shape of SeNPs was revealed by Field Emission Scanning Electron Microscopy (FE-SEM) examination (EVO SEM MA 15/18, Carl-Zeiss, Germany). The energy dispersive X-ray spectroscopy (EDX) was performed using a Bruker EDX spectrometer to determine the elemental composition of the SeNPs.

Preparation of priming solution and seed priming method

The green synthesized SeNPs were used for seed priming and germination test. Different concentrations of SeNPs (20μM and 25μM) were prepared by dispersing NPs in distilled water via sonication for 40 min, designated as SeNP 20 and SeNP 25 respectively, followed by preparation of two concentrations Na_2SeO_3 (10μM and 20μM) by dissolving salt in distilled water, designated as Se 10 and Se 20 respectively. The concentrations were earlier optimized by screening for concentration gradient in a control plant. Healthy and uniform rice (*Oryza sativa* L.) seeds were selected and were first with running tap water Subsequently seeds were surface sterilized with 2% of sodium hypochlorite for 10 min then washed with distilled water several times. Sterilized seeds were then soaked in different concentrations of T2- Se 10, T3- Se 20, T5- SeNP 20 and T5- SeNP 25 solution

respectively for 24 h, and surface-dried on paper towel. Seeds were dried back to their original moisture content at room temperature (25 ± 2 °C), sealed in polythene bags and stored at 4 °C until further use. Seeds soaked in distilled water, were defined as hydro-priming and designated as control.

Germination test, Seedling vigor and Biochemical assay

All the tests on germination were performed in triplicates. Whatman filter paper was placed on sterilized Petri plates. Ten primed seeds of each treatment were placed in their respective labelled Petri plate, and then moistened with 5 mL of distilled water. All the Petri plates were kept in an incubator under dark condition at 30° in the BOD. After 24 h of incubation, germination tests for, amylase activity, total soluble sugar content and starch content were performed and repeated upto 96 h at the interval of 24 h. Simultaneously, the seeds were allowed to germinate and the germination was monitored for next 7 days till 100% germination achieved in control. When the radicle length of germinating seeds extended up to 5 mm in length then it was considered as germinated and data on germination percentage (G%) was calculated according to the previous literatures (Ellis and Roberts, 1981; Feizi et al., 2013).

2,3,5-Triphenyltetrazolium Chloride (TTC) Staining

TTC staining is a frequently used method to determine the viability of cells or tissues. TTC is a white salt and substrate of dehydrogenases and its metabolite is a red dye, which turns the cell red in viable tissue and white in dead tissue. After incubation in water, SeNPs and Se salt for 24, 48, 72 and 96 h five whole seeds with embryo were selected from each treatment and stained with 0.5% TTC at 35ºC for 3 h and then washed five times with distilled water and photographed under a SteREOLumarV12 stereomicroscope (R. Li et al., 2017; Soares et al., 2016).

Starch metabolism and starch agar plate α-amylase assay

After germination starch metabolism in germinating seeds were determined by measuring α-amylase activity, total soluble sugar content and starch content upto 96 h at the interval 24 h. The activity of α- amylase enzyme was assayed from the saccharifying activity as per the protocol described by Bernfeld (1955). The enzyme activity calculated by the formula

given by Yaldagard et al. (2008). For confirming the effect nanopriming on α-amylase activity, starch-agar plate assay (Qualitative assessment) was performed following the protocol of Chen et al. (2006). The embryo-less half-seeds were sterilized and placed perpendicularly on starch-agar plates [2% agar plate containing 0.2 % soluble potato starch, 10 mM sodium acetate and 2 mM CaCl2 (pH 5.3)] and incubated at room temperature in the dark for and observed for 3 days. 1 μM gibberellic acid (GA3) was added to cooled agar that acted as positive control (GA+), while plate having no GA3 was defined as negative control (GA-). After 3 days of incubation, embryo-less seeds were removed and staining agar plate with IKI solution for testing the α-amylase production. Clear zones (transparent halos) appear if α-amylases are synthesized in endosperms and secreted into the starch agar, resulting in starch hydrolysis. The experiment was performed in triplicates. Total soluble sugars (TSS) were estimated using anthrone reagent as per the procedure outlined by Dubois et al. (1956). Starch content was estimated using the method described by Hodge and Hofreiter (1962). The amount of sugar was determined using the standard curve prepared from glucose.

Antioxidant enzyme assay

Nitroblue tetrazolium (NBT) photochemical assay was used to estimate the activity of superoxide dismutase (SOD) (Beyer and Fridovich, 1987). After 24 h of imbibition, 500 mg seeds were grinded and mixed with 100 mM potassium phosphate buffer (PPB) (pH 7.8) containing 1 % polyvinyl-pyrrolidone (PVP), 0.1 mM ethylene-diamine-tetra acetic acid (EDTA) and 0.5 % Triton X-100. Followed by centrifugation at 35,000 *g* for 15 min at 4 ◦C and supernatant was collected. The resultant supernatant was used to detect antioxidant enzyme activity. For SOD, 500 μL of supernatant was mixed with 4 mL solution containing 20 mM methionine, 0.15 mM EDTA and 0.12 mM NBT. Exposure to fluorescent lamps for 30 min was given to tubes containing samples whereas the sample labelled as blank was kept in the dark. One unit of the enzyme activity was defined as the quantity of enzyme required to result in a 50 % inhibition of the rate of NBT reduction at 560 nm. Activity of catalase (CAT) was anticipated in terms of the dissociation of H2O2 at 240 nm for 1 min (extinction coefficient of 39.4 mM□ 1 cm□ 1) according to Aebi (1984). 2 mL of reaction mixture contained 200 μL enzyme extract, 50 mM PPB(pH 7.0), 10 mM

H2O2 and 1 mM EDTA. 1 n mol H2O2 dissociated min□ 1 defined as reactivity of one unit of enzyme. Activity of ascorbate peroxidase (APX) was estimated according to Chen and Asada (1989). Decreased absorbance was monitored at 290 nm over a period of 4 min. For this the reaction mixture comprised 500 μL of enzyme extract, 50 mM PPB (pH 7.0), 0.5 mM ascorbate and 0.2 mM H2O2. The activity was calculated using an extinction coefficient of 2.8 mM- 1 cm- 1.

Histochemical localization in seeds

The accumulation of ROS (H_2O_2 and O_2^-) were localized using 3,3-diaminobenzidine (DAB) and nitro-blue tetrazolium (NBT) respectively whereas the production and accumulation of •OH, can indirectly reflect the peroxidase activity which was detected histochemically by TMB staining as described by (Chen et al.,2016 and Li et al., 2017b) in seeds. For H_2O_2 and O_2^- five Seeds from each treatments being soaked for different intervals (24, 48 h and 72 h) were collected and incubated again in 1 mg ml-1 of DAB stain solution and 1 mM NBT in 10 mM Tris-HCl respectively for 30 min at room temperature. For TMB staining seeds soaked for different intervals (24, 48 h and 72 h) from each treatment were selected and incubated in 0.2% (w/v) TMB and 1 mM H2O2 in 20 mM phosphate buffer (pH 6.5) at room temperature for 30 min. Finally all the stained seeds were photographed with the SteREOLumarV12 stereomicroscope.

Statistical Analysis

The data obtained from four different replicates are analysed for Analysis of variance (ANOVA), Duncan's multiple range test (DMRT) post-hoc analysis and correlation analysis by using software R (v 4.2.1). UV, XRD, Raman and FT-IR graphs were generated using Origin v2022b. Other graphs are generated using Graphpad Prism (v8.0.2).

Table 1 Preliminary qualitative screening analysis of *Vitis vinifera* L. extract				
**S. No.**	**Phytochemicals**	**Reagents/ Test**	**Result**	**Reference**
1	Alkaloids	1. Dragendorff's reagent	Positive	Ekwueme et al., 2015
		2. Hager's reagent	Positive	Harborne, 1973
		3. Wagner's reagent	Positive	Ekwueme et al., 2015
2	Terpenoids	1. Salkowski's reagent	Positive	Tiwari et al., 2011
		2. Chloroform and Sulphuric acid	Positive	Panchal et al., 2021
3	Steroids	1. Sulphuric acid	Negative	Ekwueme et al., 2015
		2. Libermann-Buchard test	Negative	Hossain et al., 2013
4	Phenols	1. Ferric chloride	Positive	Ali et al., 2017
5	Saponins	1. Foaming with distilled water	Positive	Okerulu et al., 2017
		2. Emulsion test with the olive oil	Positive	Hashmi et al., 2021
6	Flavonoids	1. Alkaline reagent (NaOH)	Positive	Sajid et et al., 2016
		2. Ferric chloride (FeCl3)	Positive	Hashmi et al., 2021
7	Tannins	1. Alkaline reagent (NaOH)	Negative	Hashmi et al., 2021
		2. Ferric chloride (FeCl3)	Negative	Khan et al., 2012
8	Reducing Sugars	1. Fehling's solution	Positive	Harborne, 1973
9	Soluble Carbohydrates	1. Molisch reagent	Positive	Harborne, 1973
10	Proteins	1. Nitric acid	Negative	Tiwari et al., 2011
		2. Biuret test	Negative	Sharma et al., 2015
11	Antioxidants	1. DNPH Test	Positive	Sharma et al., 2015

References

Okerulu, I. O., Onyema, C. T., Onwukeme, V. I., & Ezeh, C. M. (2017). Assessment of phytochemicals, proximate and elemental composition of Pterocarpus soyauxii (Oha) leaves. American Journal of Analytical Chemistry, 8(06), 406..

Hashmi, H. F., Bibi, S., Anwar, M., & Khan, M. R. (2021). Qualitative and quantitative analysis of phytochemicals in Lepidium Pinnatifidum Ledeb. *Sch Int J Tradit Complement Med*, *4*(5), 67-75.

Ekwueme, F. N., Nwodo, O. F. C., Joshua, P. E., Nkwocha, C., & Eluka, P. E. (2015). Qualitative and quantitative phytochemical screening of the aqueous leaf extract of Senna mimosoides: Its effect in in vivo leukocyte mobilization induced by inflammatory stimulus. *Int J Curr Microbiol Appl Sci*, *4*(5), 1176-1188.

Harborne JB (1973) Phenolic compounds. In Phytochemical methods (pp. 33-88). Springer, Dordrecht

Sharma, A., Goyal, R., & Sharma, L. (2015). Potential biological efficacy of Pinus plant species against oxidative, inflammatory and microbial disorders. BMC complementary and alternative medicine, 16(1), 35.

Panchal Mital, D., & Jha, C. V. (2021). Qualitative and quantitative phytochemical screening of three plants stem bark and leaves from sapotaceae family. *Int. J. Multidiscip. Educ. Res*, *6*(8), 1-6.

Tiwari P, Kumar B, Kaur M, Kaur G, Kaur H (2011) Phytochemical screening and extraction: a review Int. J PharmSciRes 1(1):98–106

Hossain MA, AL-Raqmi KAS, AL-Mijizy ZH, Weli AM, Al-Riyami Q (2013) Study of total phenol, flavonoids contents and phytochemical screening of various leaves crude extracts of locally grown Thymus vulgaris. Asian Pac J Trop Biomed , 3(9), pp.705–710

Ali, S., Khan, M. R., & Sajid, M. (2017). Protective potential of Parrotiopsis jacquemontiana (Decne) Rehder on carbon tetrachloride induced hepatotoxicity in experimental rats. Biomedicine & Pharmacotherapy, 95, 1853-1867.

Sajid, M., Khan, M. R., Shah, N. A., Shah, S. A., Ismail, H., Younis, T., & Zahra, Z. (2016). Phytochemical, antioxidant and hepatoprotective effects of Alnus nitida bark in carbon tetrachloride challenged Sprague Dawley rats. BMC complementary and alternative medicine, 16(1), 268

Khan, R. A., Khan, M. R., Sahreen, S., & Shah, N. A. (2012). Hepatoprotective activity of Sonchus asper against carbon tetrachloride-induced injuries in male rats: a randomized controlled trial. BMC complementary and alternative medicine, 12(1), 90.

Ahmed S, Ahmad M, Swami BL, Ikram S (2016) A review on plants extract mediated synthesis of silver nanoparticles for antimicrobial applications: a green expertise. J Adv Res 7(1):17–28.

Weng X, Guo M, Luo F, Chen Z (2017) One-step green synthesis of bimetallic Fe/Ni nanoparticles by eucalyptus leaf extract: biomolecules identification, characterization and catalytic activity. Int J Chem Eng 308:904–911
